# Dermal Exposure to Jet Fuel JP-8 Significantly Contributes to the Production of Urinary Naphthols in Fuel-Cell Maintenance Workers

**DOI:** 10.1289/ehp.8288

**Published:** 2005-09-29

**Authors:** Yi-Chun E. Chao, Lawrence L. Kupper, Berrin Serdar, Peter P. Egeghy, Stephen M. Rappaport, Leena A. Nylander-French

**Affiliations:** 1 Department of Environmental Sciences and Engineering, and; 2 Department of Biostatistics, School of Public Health, University of North Carolina at Chapel Hill, Chapel Hill, North Carolina, USA

**Keywords:** 1-naphthol, 2-naphthol, biomarker, dermal exposure, jet fuel (JP-8), naphthalene (CAS 91-20-3), Pratt index, relative contribution, tape stripping, total body dose

## Abstract

Jet propulsion fuel 8 (JP-8) is the major jet fuel used worldwide and has been recognized as a major source of chemical exposure, both inhalation and dermal, for fuel-cell maintenance workers. We investigated the contributions of dermal and inhalation exposure to JP-8 to the total body dose of U.S. Air Force fuel-cell maintenance workers using naphthalene as a surrogate for JP-8 exposure. Dermal, breathing zone, and exhaled breath measurements of naphthalene were obtained using tape-strip sampling, passive monitoring, and glass bulbs, respectively. Levels of urinary 1- and 2-naphthols were determined in urine samples and used as biomarkers of JP-8 exposure. Multiple linear regression analyses were conducted to investigate the relative contributions of dermal and inhalation exposure to JP-8, and demographic and work-related covariates, to the levels of urinary naphthols. Our results show that both inhalation exposure and smoking significantly contributed to urinary 1-naphthol levels. The contribution of dermal exposure was significantly associated with levels of urinary 2-naphthol but not with urinary 1-naphthol among fuel-cell maintenance workers who wore supplied-air respirators. We conclude that dermal exposure to JP-8 significantly contributes to the systemic dose and affects the levels of urinary naphthalene metabolites. Future work on dermal xenobiotic metabolism and toxicokinetic studies are warranted in order to gain additional knowledge on naphthalene metabolism in the skin and the contribution to systemic exposure.

Jet propulsion fuel 8 (JP-8) has been recognized as a major source of chemical exposure for fuel-cell maintenance workers [Agency for Toxic Substances and Disease Registry (ATSDR) 1998; National Research Council 2003]. Despite the increasing number of studies conducted on JP-8 (Carlton and Smith 2000; Egeghy et al. 2003; Pleil et al. 2000; Rhodes et al. 2003; Serdar et al. 2003), knowledge of JP-8 exposure, uptake, metabolism, and its potential effects on human health is limited, and only one study has been published on the quantification and assessment of JP-8 dermal exposure (Chao et al. 2005). Previous studies on JP-8 exposure have emphasized the potential contribution of dermal exposure to increased biomarker levels (i.e., urinary 1- and 2-naphthol) based on surrogate factors used as indicators of dermal exposure (e.g., skin irritation, work inside the fuel cell, cleaning fuel puddles) (Egeghy et al. 2003; Serdar et al. 2004). To our knowledge, exposure to JP-8 and the contributions of the different exposure routes (i.e., inhalation and dermal) to the total body dose have not been quantitatively investigated.

In this study, we examined the contributions of dermal and inhalation exposure to naphthalene, as a marker for JP-8 exposure (Chao et al. 2005; Chao and Nylander-French 2004; Egeghy et al. 2003; Serdar et al. 2003, 2004), to the levels of urinary 1-naphthol and 2-naphthol in U.S. Air Force (USAF) fuel-cell maintenance workers. We demonstrate, using multiple linear regression analyses, that both dermal and inhalation exposure to JP-8 significantly contribute to urinary 1-naphthol and 2-naphthol levels. The relative contributions of both exposure routes to the total body dose were also estimated.

## Materials and Methods

### Study population.

This study was conducted at six USAF bases, as a part of a broader project (Institute of Environmental and Human Health 2001), to investigate the relative contributions of JP-8 exposures through different routes (i.e., inhalation and dermal) to the total body dose of fuel-cell maintenance workers. Workers were recruited with informed consent from active-duty USAF personnel who routinely worked with, or were exposed to, JP-8. Although 339 USAF personnel were enrolled in the overall project, a total of 85 fuel-cell maintenance workers were included in our particular study. Approval for human subject use was obtained from the institutional review board for each of the participating investigators and for the USAF, and the study complied with all applicable U.S. requirements and regulations.

Questionnaires were collected after the work shift to obtain information on demographic factors, including job tasks, the use of personal protective equipment, smoking status, and other work-related characteristics. Work diaries for each individual were also recorded during the survey, including detailed information on work tasks, task durations, and the use of personal protective equipment. Workers included in this study entered fuel cells during the day sampling was conducted. They performed maintenance work inside the fuel cell and thus were expected to have the highest potential for both dermal and inhalation exposure to JP-8. To reduce inhalation exposure, all workers wore air-supplied respirators when working inside the fuel cell. Although USAF personnel had been assigned *a priori* into exposure groups (Egeghy et al. 2003; Serdar et al. 2004), we note that these categories did not fully correspond with the work scenarios given in the work diaries on the day of investigation. Therefore, in the present investigation we relied upon the work diaries to define job tasks and work scenarios.

### Sample collection and analysis.

#### Dermal samples.

Collection and analyses of tape-strip samples have been described previously (Chao et al. 2005). Briefly, for each worker, three body regions with potentially the greatest JP-8 exposure were selected for dermal sampling. Tape-strip samples were collected with Cover-Roll (Beiersdorf AG, Germany) tape at each exposed body location, three locations total, after the work shift. For each worker, body regions sampled included three of the following body parts: forehead, neck, shoulders, arms, hands, legs, knees, feet, and buttocks. Samples were analyzed by gas chromatography–mass spectrometry (GC-MS) (Chao and Nylander-French 2004). The amount of naphthalene removed with the three successive tape-strip samples was adjusted for the surface area of the particular region sampled in order to estimate the regional dermal exposure to naphthalene. For each worker, the regional surface areas were estimated by the Lund and Browder chart (Deitch 1999) and by Haycock’s formula (Haycock et al. 1978). The whole-body dermal exposure (nanograms per square meter) was calculated by summing the estimated regional dermal naphthalene concentrations of the three sampled regions (e.g., arm, neck, and leg) and by conservatively assuming that no exposure to the other unsampled regions occurred.

#### Breathing-zone, breath, and urine samples.

Personal inhalation exposure to naphthalene was monitored during the 4-hr work shift with passive monitors attached to the workers’ shirt collars. Exhaled-breath samples were collected using 75-cm^3^ glass bulbs before and after the work shift inside the hangar (Egeghy et al. 2003). Sampling and analyses of breathing-zone and breath samples have been described previously (Egeghy et al. 2003). Briefly, breath samples were passively transferred from the glass bulbs to Tenax (SKC Inc., Eighty Four, PA) tubes before analysis. Both breathing-zone air and breath samples were analyzed by thermal desorption followed by GC-MS with photo ionization detection.

Both 1-naphthol and 2-naphthol concentrations were determined from urine samples collected from each worker before and after the work shift. Collection and analyses of urine samples have been described elsewhere (Serdar et al. 2003). Briefly, 2 mL urine was brought to room temperature and 50 μL hexane solution containing 1 μg/mL 1-naphthol-d_7_ (internal standard) was added. The sample was hydrolyzed with β-glucuronidase/sulfatase and extracted twice with a total of 7 mL ethyl acetate. After evaporation under nitrogen, the residue was derivatized with Tri-Sil TBT (Pierce, Rockford, IL) in hexane. The trimethylsilyl ether derivatives were then analyzed by GC-MS in single-ion monitoring mode.

### Statistical analyses.

All exposure data (dermal, breathing-zone air, breath, and urine) were natural log-transformed to help satisfy assumptions regarding normality and homogeneous variance. Paired analyses were performed to investigate the differences between pre- and postexposure measurements of breath naphthalene and urinary 1- and 2-naphthol levels, as well as between postexposure urinary 1- and 2-naphthol levels. Multiple linear regression analysis (Proc REG procedure in SAS, version 8.2; SAS Institute, Cary, NC) was used to investigate the contributions of JP-8 exposure (dermal and inhalation), smoking, and other covariates obtained from questionnaires to urinary 1- and 2-naphthol concentrations. Stepwise variable selection was used to determine final regression models, with inclusion and elimination decisions about predictors conducted at the α = 0.10 level. Possible collinearity problems were investigated using eigenvalue analyses and variance inflation factors. Possible outliers were examined using studentized residuals. All statistical analyses were performed using SAS software.

The multiple linear regression model structure adopted was of the general form:


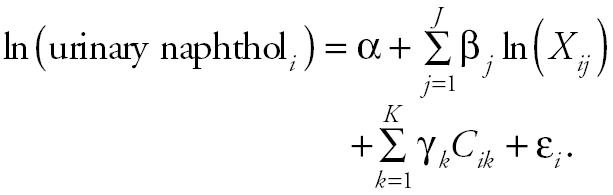


Here, the outcome variable ln(urinary naphthol*_i_*) [ln(ng/L)] is the natural logarithm of either the *i*th worker’s urinary 1-naphthol or urinary 2-naphthol level; *X_ij_* represents the *i*th worker’s *j*th exposure level to JP-8 (dermal, breathing-zone, or breath naphthalene measurement); and *C_ik_* represents the *k*th covariate value for the *i*th worker based on questionnaires providing information on smoking status (38 smokers, 47 nonsmokers), race (74 white, 11 nonwhite), sex (81 males, 4 females), job tasks [handle foams (*n* = 73), hold ventilation (*n* = 58), remove bolts (*n* = 55), remove foams (*n* = 76), remove tank door (*n* = 63)], and so forth.

The predictor variable effects consisted of α, the intercept; β*_j_*, the regression coefficient for the natural logarithm of JP-8 exposure {e.g., the natural logarithm of dermal naphthalene [ln(ng/m^2^)], breathing-zone or breath naphthalene [ln(ng/m^3^)]}; and γ*_k_* , the regression coefficient for covariate *k*. Two models were fitted using different inhalation markers. We used the breathing-zone naphthalene level in model 1 and the end-exhaled breath naphthalene level in model 2 as inhalation markers.

The relative contributions of predictor variables in the final regression models were determined by the proportionate contribution that each predictor made to the regression model multiple *R*^2^ using the Pratt index (Pratt 1987). For each predictor in a final regression model, the Pratt index for that predictor is the product of its estimated standardized regression coefficient and the simple correlation between that predictor and the outcome variable. One particularly nice property of the Pratt index is that the sum of the Pratt indices for all predictors equals *R*^2^. The Pratt index for each predictor can be rescaled by dividing it by the model *R*^2^ and multiplying by 100, so that the resulting number can be interpreted as the percentage of the model *R*^2^ accounted for by that predictor.

## Results

### Exposure measurements.

The measured dermal, breathing-zone, and breath naphthalene levels, as well as urinary 1- and 2-naphthol levels, for the 85 USAF fuel-cell maintenance workers are described in Table 1. The geometric mean (GM) [geometric standard deviation (GSD)] of dermal naphthalene level were 4,180 (9.35 ng/m^2^) with a range of 100 ng/m^2^ to 5,090 μg/m^2^. The GM (GSD) of breathing-zone naphthalene were 614,000 and (2.12 ng/m^3^) with a range of 670 ng/m^3^ to 3,910 μg/m^3^. The postexposure levels of breath naphthalene and urinary 1- and 2-naphthol were significantly higher than the preexposure levels (all *p*-values < 0.0001). In addition, postexposure urinary 2-naphthol levels were greater than postexposure urinary 1-naphthol levels (*p* < 0.0001).

### Regression analysis for urinary naphthol levels using breathing-zone naphthalene as an inhalation marker (model 1).

Model 1 explained 26.6% and 26.3% of total variance in the urinary 1- and 2-naphthol levels in entrants, respectively (Table 2). In the model for urinary 1-naphthol, breathing-zone naphthalene and smoking were the only significant predictors, explaining 88.2% and 11.8% of total variance, respectively, using the Pratt index of relative importance (Pratt 1987). For urinary 2-naphthol, dermal and breathing-zone naphthalene and smoking were significant, explaining 51.1%, 35.8%, and 13.1% of total variance, respectively. These results indicate that dermal exposure to naphthalene contributed significantly to urinary 2-naphthol levels but not to urinary 1-naphthol levels among the fuel-cell maintenance workers.

### Regression analysis for urinary naphthol levels of entrants using end-exhaled breath naphthalene as an inhalation marker (model 2).

Because the fuel-cell maintenance workers wore respiratory protection when entering the fuel cell, breathing-zone naphthalene could be regarded as an unreliable measure of personal inhalation exposure, because it most likely represents an overestimation of the personal inhalation exposure under these conditions. Thus, end-exhaled breath naphthalene measured immediately after the end of work was investigated as a potential inhalation marker in model 2. This model explained 31.8% and 30.9% of total variance in urinary 1- and 2-naphthol levels, respectively (Table 2). For urinary 1-naphthol, breath naphthalene and smoking were the only significant predictors, explaining 87.2% and 12.8% of total variance, respectively. For urinary 2-naphthol, dermal and breath naphthalene and smoking were significant predictors, explaining 32.3%, 52.9%, and 14.8% of total variance, respectively. These results also suggest that dermal exposure to naphthalene contributes significantly to urinary 2-naphthol levels but not to urinary 1-naphthol levels. Although the relative contribution of dermal naphthalene to urinary 2-naphthol levels decreased in model 2 relative to model 1, this may be attributed to the fact that breath naphthalene may actually reflect both dermal and inhalation exposure to JP-8.

## Discussion

Urinary biomarkers have been widely used for assessing exposure from all relevant exposure routes. For JP-8 exposure, urinary 1- and 2-naphthols have previously been used as biomarkers of exposure (Serdar et al. 2004). Using quantitative measures, we investigated the contributions of dermal and inhalation exposure to JP-8 to urinary naphthols levels. As expected, all postexposure measurements, including breath naphthalene and urinary 1- and 2-naphthol, were significantly greater than preexposure measurements (*p* < 0.0001). Interestingly, we also observed greater postexposure urinary 2-naphthol levels than postexposure urinary 1-naphthol levels (*p* < 0.0001). Our statistical analyses indicate that dermal exposure to JP-8 contributed significantly to urinary 2-naphthol levels but not to urinary 1-naphthol levels in both model 1 and model 2 (Table 2). This difference in findings may be due to naphthalene metabolism in the skin by mixed-function oxygenases and conjugation enzymes, which may result in a proportional difference between urinary 1- and 2-naphthol levels. Like liver, skin contains phase 1 and phase 2 enzymes, which are capable of detoxifying xenobiotics (Pendlington et al. 1994). Depending upon exposure pathway and dose, the spectrum and abundance of metabolites may change because of inductive capacity and saturation kinetics of different pathways of metabolism (Henderson et al. 1989; Lee et al. 2000). Because the spectrum of constitutive and inducible enzymes in the skin is unknown, further metabolism and toxicokinetic studies are warranted to investigate naphthalene metabolism involving various mixed-function oxygenases and conjugation enzymes and their relative spectrums. Overall, the impact of smoking on urinary 1- and 2-naphthol levels was minimal (11.8–14.8%) compared with dermal and inhalation exposure to JP-8. These results demonstrate that urinary 1- and 2-naphthol levels reflect exposure through both dermal and inhalation routes. Furthermore, these findings suggest that dermal exposure to JP-8 significantly contributed to the naphthol levels measured in urine.

Although these workers had high levels of breathing-zone naphthalene (Table 1), their true inhalation exposure was certainly lower, because all workers wore air-supplied respirators when working inside the fuel cells. The use of these respirators prevented or, at the very least, restricted inhalation exposure to JP-8 during the task with the greatest potential for both dermal and inhalation exposure. With respiratory protection, breathing-zone naphthalene can no longer be regarded as a reliable measure of inhalation exposure for entrants. Although breathing-zone naphthalene was a significant factor contributing to both urinary 1- and 2-naphthol levels in our analyses, it most likely represents an overestimation of the inhalation exposure level under these conditions. To better understand the effects of this potential overestimation of inhalation exposure due to using breathing-zone measurements, we used the end-exhaled breath naphthalene measured immediately after the end of work as a potential inhalation marker in model 2 (Table 2). The higher *R*^2^ values in model 2 using the end-exhaled breath naphthalene measurements provide some evidence as to their suitability as measures of inhalation exposure. However, we have to acknowledge that the end-exhaled breath naphthalene levels for these workers most likely reflect the contributions of both inhalation and dermal exposure routes and therefore also represent overestimations of inhalation exposure. Nevertheless, we believe that, for the types of workers investigated in this study, the end-exhaled breath naphthalene level is a better measure of inhalation exposure than is the breathing-zone naphthalene level.

One limitation of the data set we analyzed is that preexposure levels of urinary 1- and 2-naphthol were missing for roughly half of our study subjects. In this data set, smoking status is significantly associated with pre-exposure urinary naphthol levels but not with postexposure urinary naphthol levels. So, in our regression analyses, we used the dichotomous smoking status variable as a surrogate for these continuous measures of preexposure urinary naphthol levels, and we acknowledge that this is a less than optimal strategy.

In summary, we observed that dermal exposure to JP-8, along with inhalation exposure, is a major exposure route contributing significantly to the total body dose as measured by urinary 1- and 2-naphthol levels. We recommend that dermal exposure monitoring using the tape-strip technique be performed in conjunction with biologic monitoring when assessing exposure to JP-8. This is particularly important when respiratory protection is used and the potential for inhalation exposure is limited compared with dermal exposure. Personal protection actions and engineering controls are needed to reduce dermal contact with JP-8. Future studies are warranted to understand naphthalene-specific metabolism and the spectrum of naphthalene metabolites in the skin and their contribution to systemic exposure.

## Figures and Tables

**Table 1:** GMs and GSDs of dermal, breathing-zone, and breath naphthalene and urinary 1- and 2-naphthol levels observed in USAF fuel-cell maintenance workers.

Indicator of exposure	No.	GM	GSD	Minimum	Maximum
Dermal naphthalene (ng/m^2^)	85	4,180	9.35	100	5,090,000
Breathing-zone naphthalene (ng/m^3^)	83	614,000	2.21	670	3,910,000
Preexposure breath naphthalene (ng/m^3^)	82	492	1.99	330	16,100
Breath naphthalene (ng/m^3^)	72	9,230^a^	2.88	667	75,800
Preexposure urinary 1-naphthol (ng/L)	43	4,200	3.77	242	39,000
Urinary 1-naphthol (ng/L)	85	28,000^b^	2.26	483	127,000
Preexposure urinary 2-naphthol (ng/L)	43	4,350	3.06	424	37,900
Urinary 2-naphthol (ng/L)	85	38,400^c^,^d^	2.46	485	315,000

All statistical tests were performed on log-transformed data.

a Significantly different from preexposure breath naphthalene levels (*p* < 0.0001).

b Significantly different from preexposure urinary 1-naphthol levels (*p* < 0.0001).

c Significantly different from preexposure urinary 2-naphthol levels (*p* < 0.0001).

d Significantly higher than urinary 1-naphthol levels (*p* < 0.0001).

**Table 2:** Regression analyses for urinary 1- and 2-naphthol levels of fuel-cell maintenance workers when either breathing-zone or end-exhaled breath naphthalene level was used as an inhalation marker.

Urinary metabolite	No.	*R*^2^	Predictor	Parameter estimate	SE	*p*-Value^a^	Relative contribution (%)^b^
1-Naphthol	83^c^	0.27	Intercept	3.48	1.32	0.0101	
			ln(breathing-zone naphthalene)	0.50	0.10	< 0.0001	88.2
			Smoking (0 = no, 1 = yes)	0.28	0.16	0.0808	11.8
	72^d^	0.32	Intercept	6.14	0.75	< 0.0001	
			ln(end-exhaled breath naphthalene)	0.43	0.08	< 0.0001	87.2
			Smoking (0 = no, 1 = yes)	0.36	0.17	0.0399	12.8
2-Naphthol	83^c^	0.26	Intercept	5.11	1.53	0.0013	
			ln(breathing-zone naphthalene)	0.33	0.13	0.0114	51.1
			ln(dermal naphthalene)	0.11	0.04	0.0119	35.8
			Smoking (0 = no, 1 = yes)	0.34	0.18	0.0603	13.1
	72^d^	0.31	Intercept	6.80	0.87	< 0.0001	
			ln(end-exhaled breath naphthalene)	0.30	0.12	0.0128	52.9
			ln(dermal naphthalene)	0.10	0.05	0.0790	32.3
			Smoking (0 = no, 1 = yes)	0.45	0.19	0.0238	14.8

a Stepwise regression variable inclusion and elimination decisions conducted at the α = 0.10 level.

b Estimated using the Pratt index (Pratt 1987).

c Model 1: breathing-zone naphthalene used as an inhalation marker.

d Model 2: end-exhaled breath naphthalene used as an inhalation marker.
